# Development and performance evaluation of a novel immunofluorescence chromatographic assay for histidine-rich protein 2 of *Plasmodium falciparum*

**DOI:** 10.1186/s12936-015-0740-1

**Published:** 2015-05-30

**Authors:** Keren Kang, Emmanuel E. Dzakah, Yongping Huang, Mingquan Xie, Xiaochun Luo, Wenmei Li, Jihua Wang

**Affiliations:** School of Bioscience and Bioengineering, South China University of Technology, Guangzhou, 510006 Guangdong China; National Engineering Laboratory of Rapid Diagnostic Tests, Guangzhou Wondfo Biotech Co Ltd, Guangzhou, 510663 Guangdong China; Department of Molecular Biology and Biotechnology, School of Biological Sciences, College of Agriculture and Natural Sciences, University of Cape Coast, Cape Coast, Ghana

**Keywords:** *Plasmodium falciparum*, Immunofluorescence chromatographic assay, Histidine-rich protein 2, Rapid diagnostic test

## Abstract

**Background:**

The low sensitivity and specificity of *Plasmodium falciparum* diagnostic tests pose a serious health threat to people living in endemic areas. The objective of the study was to develop a rapid assay for the detection of histidine-rich protein 2 (HRP2) of *P. falciparum* in whole blood by immunofluorescence chromatographic technology.

**Methods:**

A total of 1163 positive and negative blood samples were screened. The double-antibody sandwich assay was used to establish the kit and its performance was evaluated for sensitivity, specificity, accuracy, precision, stability, and clinical effectiveness.

**Results:**

The cut-off level of detection of the kit was 25 parasites/μl. Common interfering substances in human blood specimens, such as bilirubin, triglyceride and cholesterol had no significant effect on HRP2 antigen detection. The precision of the kit was run with different concentration of standard calibrators and the values were less than 10 %. The performance of this diagnostic kit in the detection of the calibrators has shown that a shelf life of about 12 months gives a more reliable result. Among clinical samples tested, the HRP2 test kit and the reference products had good coincidence rate in a parallel experiment and this test kit had a more sensitive detecting level to the target protein than the reference kits used in this study. The specificity and sensitivity for this test were 99.6 % (800/803) and 99.7 % (1160/1163), respectively.

**Conclusions:**

A novel HRP2 immunofluorescence detection method was developed in this study. Overall performance evaluation indicated that the kit has a rapid, high sensitivity and on-spot method for detecting *P. falciparum*.

## Background

Malaria is an infection caused by the *Plasmodium* parasite that affects human health. There are over 500 million people infected with malaria with over 1.1 million deaths each year worldwide [1, 2]. Malaria has become a serious public health concern in Asia, especially in countries in Southeast Asia, and has therefore been given adequate attention by international organizations and the developed countries. With the boom in China’s economy and the growing bilateral relationship with Africa and Southeast Asia, increasing cases of malaria infection, and less effective treatment and drug resistance have been recorded [3, 4].

Among *Plasmodium* infections in people, *Plasmodium falciparum* is the most deadly to human beings. *Plasmodium falciparum* at the asexual blood stage synthesizes three kinds of histidine-rich protein (HRP): nodules-associated HRP1, soluble HRP2 and small HRP3. Common features of these HRP are high levels of histidine and the short peptide repeats of AHH nucleotides. HRP2 is the only complete protein of the three HRPs secreted from infected erythrocytes and serves as the most diagnostic marker released by *P. falciparum* [5–7].

Detection techniques of *P. falciparum* include morphological differentiation, molecular diagnostics and diagnostic immunology. Morphological microscopic examination is the gold standard for diagnosis of malaria by thick and thin blood smears [8, 9]. It is an accurate and intuitive method that helps to determine the infection type and quantity of parasites present in the blood. However, thick and thin blood examinations require professional skills and experience, demanding complex operation. Immunological diagnosis is an alternative method that relies on the specificity of antigen-antibody reaction for the detection of the parasite by applying colloidal gold method with a sensitivity of 100 parasites/μl. Polymerase chain reaction (PCR) is a more sensitive molecular diagnostic method in which the genomic deoxyribonucleic acid is extracted from infected blood samples and *P. falciparum*-specific primers are used to detect the presence or absence of the parasite. The sensitivity of the PCR method can reach as high as one parasite/μl. However, the expensive reagents, laboratory infrastructure and skilled technical staff required for its operation makes it unsuitable for application in point-of-care testing in malaria-endemic areas [10–13].

In this study, the fluorescent nanoparticle labels were applied in the double-antibody sandwich method for the development of highly sensitive, rapid and accurate *P. falciparum* HRP2 immunofluorescence used in clinical diagnosis of malaria.

## Methods

### Reagents and instruments

Anti-PfHRP2 monoclonal antibodies and PfHRP2 recombinant antigen and Finecare™ Multi-channel FIA Meter (Model Number: WF-0901/1) were provided by the National Engineering Laboratory of Rapid Diagnostic Tests of Guangzhou Biotech Co., Ltd, China. Fluorescent latex and nitrocellulose membranes were purchased from Merck, Germany. Rabbit serum immunoglobulin IgG was purchased from Scantibodies, USA and Carestart™ reference kit was from AccessBio Company, USA. Other chemical reagents were of analytical grade. NanoDrop 2000 C spectrophotometer was from Thermo Fisher, USA. High-speed refrigerated centrifuge was obtained from Hitachi, Japan. Contact spray film machine was from Imagene Technology Company, USA.

### Sample collection

From April 2013 to July 2014, a total of 1163 (816 males and 347 females) *P. falciparum* positive and negative whole blood samples from outpatient departments were collected at the Henan Centre for Disease Prevention and Control, Jiangsu Institute of Parasitic Diseases, and Guangxi Centre for Disease Prevention and Control. The mean age was 41 years (ranging between 3 and 91 years). All patients were informed of the use of their blood samples for immunodiagnostic study and all consented to participate in the study.

### *Plasmodium falciparum* sample panel (PfSP)

This panel was prepared at the Center for Disease Control, USA and consisted of aliquots of five cultured *P. falciparum* parasite lines from geographically different endemic areas. The panel was composed of five samples each with parasite densities of 200 parasites/μl and 2000 parasites/μl, and two samples with parasite density of 5000 parasites/ μl. The panel was tested against a number of commercially available HRP2- detecting and pLDH-detecting rapid diagnostic tests (RDTs).

### *Plasmodium falciparum* internal quality control panel (PfIQCP)

The panel was provided by National Engineering Laboratory of Rapid Diagnostic Tests of Guangzhou Biotech Co., Ltd, China. The panel was composed of three references with very low detection limit (L1 to L3), one precision (J), ten negatives (N1 to N10) and positive references (P1 to P10).

### Principle

**Fig. 1:**
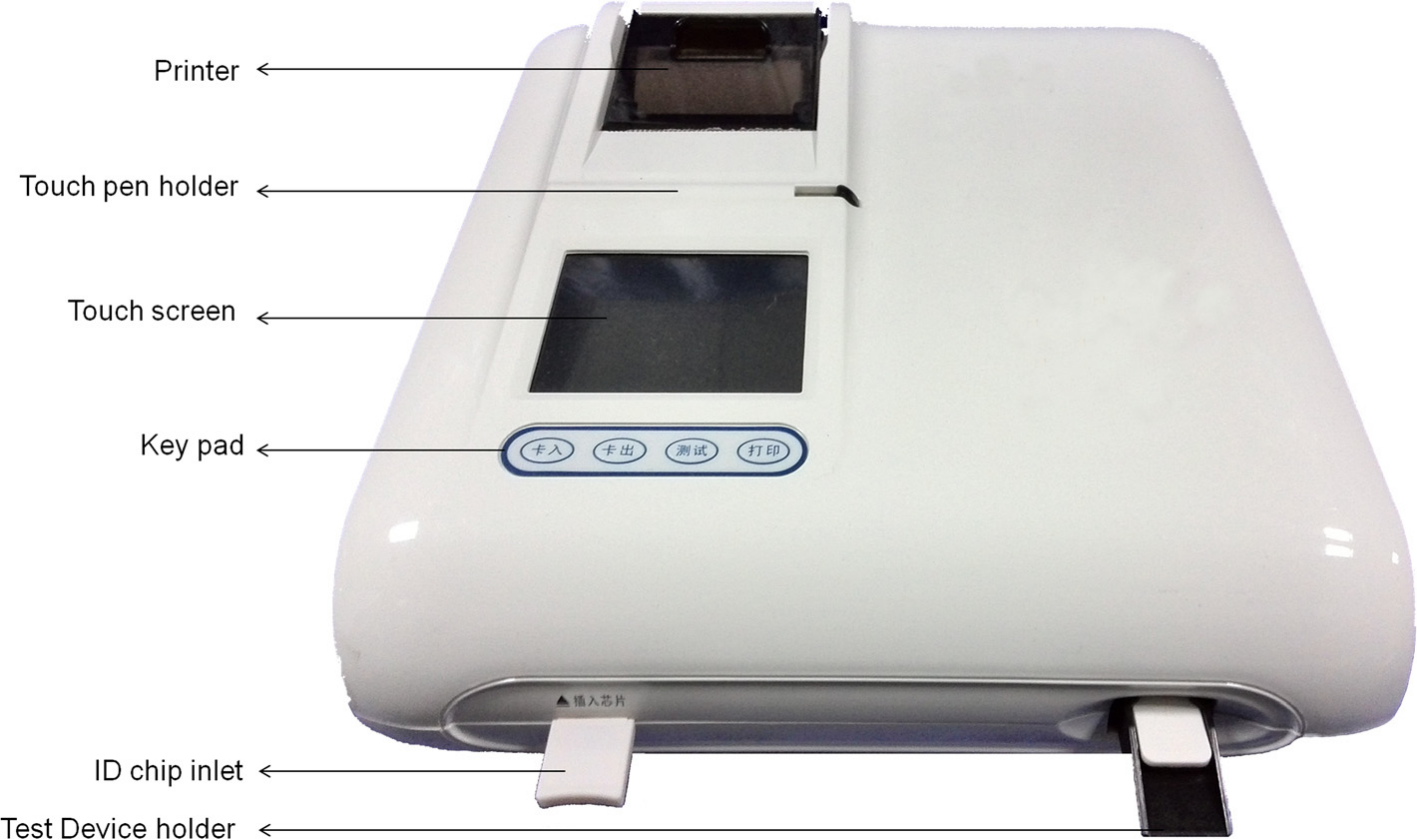
FinecareTM FIA meter. The Finecare™ FIA Meter is a portable instrument for fluorescence detection of various analytes in blood or urine. The meter equipped with a built-in test cassette holder and printer

After a buffer-mixed sample is applied to the test device, it is then inserted into Finecare™ FIA Meter and the concentration of the analyte is calculated by a pre-programmed calibration process (Fig. 1). The FIA Meter can only accept test cassette that is designed specifically for use with this instrument. The FIA Meter is equipped with a built-in test cassette holder and does not require an external holder to place the cassette in. In this study, the double-antibody sandwich assay was applied to detect PfHRP2 protein in whole blood samples. The nitrocellulose membrane used in the preparation of the test strip was coated with a buffer containing an anti-HRP2 monoclonal antibody. Whole blood samples were first mixed with erythrocyte lysis buffer containing fluorescent, nanoparticle-labelled, anti-HRP2 monoclonal antibody for 30 sec to release the HRP2 protein from the cells and to allow time for the antigen-antibody complex formation. Then the mixed sample was added drop wise to the sample well of the test cassette and allowed to stand for 15 min. The reaction mixture moved along the nitrocellulose membrane and reacted with the anti-HRP2 antibody coated at the Test Zone (T zone) on the nitrocellulose membranes, forming an antibody-antigen sandwich complex. Whole blood samples containing the HRP2 antigen accumulated more reaction complexes at the test line and hence produced a stronger fluorescence signal that reflected the quantity of HRP2 antigens present in the sample. The absence of the HRP2 antigen in the sample resulted in the absence of a detectable fluorescent signal in the T zone. C zone is the control zone and should have exhibited the fluorescence signal no matter the type of antigen existing in the specimen.

### Preparation of fluorescent nanoparticle-labelled antibodies

Fluorescent latex microspheres were dissolved in phosphate buffer and an amount of light thio N- phthalimide shot glass and carbodiimide were added. The mixture placed in an ultrasonic bath at 200 W for 30 sec at room temperature. It was incubated in the dark for 30 min and then centrifuged. The precipitate was dissolved in citric acid buffer. The fluorescent latex activated anti-HRP2 monoclonal antibody was labelled as follows: 1 ml activated latex and 0.5 mg protein were mixed thoroughly. The reaction mixture was then stirred at room temperature for 2 hrs, and washed three times with phosphate buffer by centrifugation, and the precipitate was dissolved with a phosphate buffer solution and then stored at 4 °C.

### Preparation of working buffer

Fluorescent, nanoparticle-labelled, anti-HRP2 monoclonal antibody and the phosphate dilution buffer were mixed in the ratio of 1:100 and then stored at 4 °C.

### Preparation of erythrocyte lysis buffer

About 8 g of ammonium chloride (0.15 M), 1 g of potassium bicarbonate (10 mM), and 0.37 g of disodium ethylene diamine tetra acetic acid (1 mM) were weighed and dissolved in 980 ml of triple-distilled water. The resultant solution was neutralized with 1 N hydrochloric acid or 1 N sodium hydroxide to adjust the pH: 6–6.4 and top up with triple-distilled water to a final volume of 1000 ml. The prepared buffer was stored at room temperature.

### Preparation of test card

Coating buffer containing 1 mg/ml of HRP2 monoclonal antibody and anti-rabbit IgG was sprayed onto a nitrocellulose membrane corresponding to the T zone and C zone, respectively. The membrane was allowed to dry overnight at room temperature. The nitrocellulose membrane, sample pad and absorbent paper coated with fluorescent antibody were assembled into a test cassette and stored at 4–30 °C.

### Test kit performance evaluation

The test kit performance was based on CFDA [14]. The performance evaluation parameters include the detection limit, sensitivity, specificity, stability, precision, clinical compliance rate, and other indicators.

### Cut-off values determination

About 30 HRP2 negative whole blood specimens were thoroughly mixed with erythrocyte lysis buffer before 5 μl of sample was added to the sample well followed by the addition of 75 μl of buffer to test cassette and then scanning with the Finecare™ Multi-channel FIA Meter. The signal ratio of the test zone to control zone (T zone/C zone) gives the detection value of each specimen and the mean value $\left(\overline{X_B}\right)$ and the standard deviation (SD) of the mean value (S_B_) for the 30 specimens were computed. The formula $\overline{X_B}$ +3S_B_ was used as the cut-off value for the developed kit. The density of detected *P. falciparum* was obtained by diluting the six positive samples of known parasite density, sample panel with negative whole blood and formulated as follows: 200, 100, 50, 25, 12, and 6 parasite/μl, two batches of reagents were developed to test the limit of detection of parasite. In the detection process, if the detected value is greater than the cut-off value, then the FIA Meter value is designated as ‘positive’; if it is less than the cut-off value, then the FIA Meter value is ‘negative’.

### Precision

Two batches of *P. falciparum* fluorescent detection kit were compared with PfSP to test the precision of these reference materials. Tests in each batch were repeated ten times, and the intra-assay coefficient of variation (CV) between batches was computed; CV < 10 % was considered as acceptable and indicated that the test kits were of high precision.

### Interference test

Negative samples containing bilirubin (2 g/L), triglycerides (3 g/L), cholesterol (15 g/L), and rheumatoid factor (320 IU/ml), as well as positive reference samples were tested. Test results required both positive and negative coincidence rate at 100 % to indicate that other external parameters do not affect the performance of reagents.

### Cross-reaction trial

Cross-reaction tests were performed on other clinical samples, such as non-*P. falciparum* species of malaria (*Plasmodium ovale, Plasmodium malariae* and *Plasmodium vivax*), and other viral infections, including hepatitis B and C, human immunodeficiency virus and syphilis. Ten samples each of these clinical samples were randomly selected and tested.

### Stability test

The stability test was developed to assess the stability of the kit. The test strips were incubated at 50 °C for one month and the strips were tested every week using the internal quality control panels **(**IQCP) to assess the stability at high temperature. The IQCP included the L1, L1 - L3, J, N1 - N10, and P1 - P10 panels as described earlier.

### Clinical evaluation

The developed test kit was applied in the evaluation of 1163 whole blood samples obtained from the outpatient department. Test results were compared with other commercially available reference RDTs. Any inconsistency in the results obtained was further confirmed by microscopic examination. The sensitivity and specificity of this developed kit to *P. falciparum* and non-*P. falciparum* species were assessed.

### Statistical analysis

The positive and the negative coincidence rates of the kit were computed as follows: positive coincidence rate (%) = a/(a + c) × 100, and negative coincidence rate (%) = d/(b + d) × 100; where ‘a’ is the number of positive sample in both developed and reference tests; ‘b’ is the positive samples in the developed test reagent, but with a negative reference test; ‘c’ is when the developed test is negative and the reference test positive; and, ‘d’ is also negative for both tests. Compliance rate (%) = (a + d)/(a + b + c + d) × 100.

## Results

### Cut-off value of the kit and the detection limit of parasite densities

**Fig. 2:**
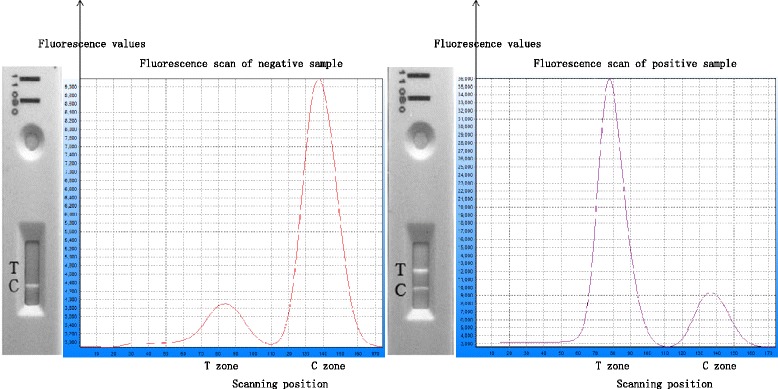
Testing of negative and positive HRP 2 samples. **a** and **b** Negative HRP2 sample test. This represents the test cassette in fluorescence excitation mode in the testing of a negative HRP2 sample (**a**). The absence of no target antigen in the sample is represented by the “T zone” having no excitation peak and the “C zone” showed a fluorescence peak (**b**). **c** and **d** Positive HRP2 sample test. The test cassette showed band detections at both T and C zones for positive samples (**c**) and the excitation in these regions are detected by the FIA and represented by the two peaks at T and C zones

There were 30 negative whole blood specimens to be tested and the average value was 0.45 with SD of 0.12. With reference to technical specifications of *in vitro* diagnostic kit product, the average detection value plus three times the SD was regarded as the detection threshold value, which is equal to 0.8 for this test. When the detected value was greater than the cut-off value, the test was designated as ‘positive’, and if it was less than the cut-off value, the test was designated as ‘negative’. Hence, to obtain the corresponding value of the detected parasite density, PfIQCP with known parasite density were serially diluted for testing. Test results showed lowest limit of detection of about 25 parasites/μl (Table 1, Fig. 2).

**Table 1:** Screening of *Plasmodium falciparum* control sample panel

Samples	Derivation of sample	Results	S/CO
Benin I 200	Benin, Africa	Positive	3.14
Benin I 2000	Benin, Africa	Positive	9.12
Santa Lucia 200	El Salvador, Central America	Positive	3.06
Santa Lucia 2000	El Salvador, Central America	Positive	6.57
Nigeria XII 200	Nigeria, Africa	Positive	3.52
Nigeria XII 2000	Nigeria, Africa	Positive	7.76
FC27/A3 200	Papua New Guinea, Australia	Positive	2.83
FC27/A3 2000	Papua New Guinea, Australia	Positive	5.98
PH1 200	Philippines, Southeast Asia	Positive	2.54
PH1 2000	Philippines, Southeast Asia	Positive	4.72

S/CO indicates the ratio of detected value to the cut-off value. S/CO < 1 is negative, S/CO ≧ 1 is positive. The greater the S/CO values, the higher the HRP2 concentration

### Precision result

In the assessment of the development of this diagnostic test, precision experiment was carried out using the precision reference of PfSP to determine the positivity of the assay, intra-assay CV < 10 % and inter-assay CV < 10 % in line with the testing requirements for a good test kit (Table 2).

**Table 2:** Precision performance of the immunofluorescence test kit

Time	Lot 1		Lot 2		Lot with lot CV (%)
	Result	S/CO	Result	S/CO	
1	Positive	1.75	Positive	1.81	6.04
2	Positive	1.69	Positive	1.89	
3	Positive	1.60	Positive	1.98	
4	Positive	1.70	Positive	1.91	
5	Positive	1.73	Positive	1.88	
6	Positive	1.69	Positive	1.95	
7	Positive	1.78	Positive	1.85	
8	Positive	1.66	Positive	1.87	
9	Positive	1.77	Positive	1.71	
10	Positive	1.64	Positive	1.82	
Within lot CV (%)	3.39		4.10		

S/CO indicates the ratio of detected value to the cut-off value. S/CO < 1 is negative, S/CO > 1 is positive. The greater the S/CO values, the higher the HRP2 concentration

Each sample was tested ten times and based on the ratio of detected value to the cut-off (S/CO) value of each test, the CV (CV = SD/mean × 100 %) within lot 1 alone and lot 2 alone were 3.39 and 4.10 %, respectively, and by lot with lot comparison was 6.04 % (Table 2).

### Interfering substances

In this study, the negative reference samples with high concentrations of bilirubin, triglycerides, cholesterol, and rheumatoid factor were tested and the positive and negative coincidence rates were 100 %, which indicated that these common contaminants did not interfere with the test results (Table 3).

**Table 3:** Performance of test kit on interfering substances

Interfering substance	Conc.	Cases	Negative reference samples		Positive reference samples	
			Criterion	Results	Criterion	Results
Bilirubin	2 g/L	20	10/10−/−	10/10−/−	10/10+/+	10/10+/+
Triglycerides	30 g/L	20	10/10−/−	10/10−/−	10/10+/+	10/10+/+
Cholesterol	15 g/L	20	10/10−/−	10/10−/−	10/10+/+	10/10+/+
Rheumatoid factor	320 IU/ml	20	10/10−/−	10/10−/−	10/10+/+	10/10+/+

10/10−/− indicates that all negative samples were tested negative for ten repeated times, and 10/10+/+ indicates that all positive samples were tested positive for ten repeated times

### Cross-reactivity

Specimens of *P. vivax*, *P. malariae*, *P. ovale* and other common clinical diseases that may impact the diagnosis of *P. falciparum* were tested and results showed that both negative and positive samples did not interfere with the diagnosis of *P. falciparum* (Table 4).

**Table 4:** Cross-reactivity determination

Sample type		Results
Malaria samples	*P. malariae*	Negative (4/4−/−)
	*P. vivax*	Negative (10/10−/−)
	*P. ovale*	Negative (10/10−/−)
Hepatitis B virus positive specimens		Negative (10/10−/−)
Hepatitis C virus positive specimens		Negative (10/10−/−)
Human Immunodeficiency Virus positive specimens		Negative (10/10−/−)
*Treponema pallidum* positive specimens		Negative (10/10−/−)

4/4−/− indicates that four cases of *P. malariae* specimens were tested negative; 10/10−/− indicates that ten cases corresponding cross-reactive specimens were tested negative

### Stability test

To ascertain the shelf life of this reagent, stability studies were carried out on the newly developed test strips at 50 °C for 1, 7, 15, 22, and 30 days and then tested with PfIQCP. The results showed that the duration of storage at this high temperature does not have any significant effect on the minimum limit of detection, and precision detection of both negative and positive reference samples (Table 5). The shelf life of this test kit was estimated to be at least 12 months.

**Table 5:** Stability test and analysis

Days	Internal quality control panel					
	Positive reference samples (low concentration)			Precision reference samples	Negative reference samples	Positive reference samples
	L_1_	L_2_	L_3_	(J)	N_1_ to N_10_	P_1_ to P_10_
1	−	+	+	10/10+/+	10/10−/−	10/10+/+
7	−	+	+	10/10+/+	10/10−/−	10/10+/+
15	−	+	+	10/10+/+	10/10−/−	10/10+/+
22	−	+	+	10/10+/+	10/10−/−	10/10+/+
30	−	+	+	10/10+/+	10/10−/−	10/10+/+

“+” indicates positive; “-” indicates negative

### Clinical test

A total of 1163 whole blood clinical samples were tested in multiples and parallel design. Test results were compared with those obtained from reference diagnostic reagents. Inconsistent results were further confirmed by the microscopic examination gold standard. The results showed that this *P. falciparum* HRP2 fluorescence diagnostic kit had a 100 % (360/360) detection rate for positive samples whereas the Carestart colloidal gold test kit. The specificity was 99.6 % (800/803) for the negative samples and a sensitivity of 99.7 % (1160/1163) was observed for all positive samples tested (Table 6).

**Table 6:** Fluorescent HRP2 diagnostic test and reference kit in the screening of clinical samples

		Developed kit		Total
		Positive	Negative	
Reference kit	Positive	360	0	360
	Negative	3	800	803
	Total	363	800	1,163

Compared with the reference kit, the positive coincidence rate of the two kits is 100 %(360/360), and the negative coincidence rate is 99.62 % (800/803)

## Discussion

Malaria is a curable infectious disease affecting millions of people worldwide. A rapid, sensitive and accurate diagnosis could prevent the spread of disease in malaria-endemic areas and guide clinical treatment for the use of anti-malarial drugs. The immunofluorescence chromatographic technology described here is a technique for the diagnosis of *P. falciparum* HRP2.

HRP2 is specific to *P. falciparum,* which is an abundant water-soluble protein with good thermal stability. It is present in the cytoplasm of infected cells and is the World Health Organization’s (WHO) recommended diagnostic antigen for *P. falciparum* [8]. The conventional PfHRP2 rapid diagnostic methods are mainly based on labelled colloidal gold immunochromatography and interpreted by the naked eye. The sensitivity of these diagnostic tests is approximately 100 parasites/μl and the low sensitivity limitations make current reagents ineffective in the diagnosis of malaria. In order to help resolve this limitation, it is important to develop a novel technique to improve the performance of the sensitivity and specificity of RDTs.

Immunofluorescence chromatographic assay had been used in recent years to detect the presence of HRP2 that are in very small amounts in human blood or urine [15]. Fluorescence-based immunochromatographic assays that employ conjugates of fluorescent microspheres and monoclonal antibodies for detection has been reported and suggestions of extending this technique to chemical contaminants and antigen detection among others have been recommended [16–18]. Similarly, a quantitative analysis of prostate-specific antigen (PSA) in human blood serum samples by fluorescence immunochromatography using anti-PSA monoclonal antibodies had been demonstrated earlier [19].

The relatively high sensitivity that characterizes the use of the immunofluorescence chromatography for the detection of HRP2 in malaria samples makes it an alternative for immunodiagnosis of the disease. This study established a highly sensitive, rapid diagnostic reagent based on the use of fluorescent latex particle-labelled HRP2 antibody as a detection antibody and solid-phase specific antibody that serves as an HRP2 capture antibody. The fluorescent signal was detected by a portable FIA meter which uses an LED as the excitation light source. The emitted light from the fluorescence dye is collected and converted into an electrical signal and outputs. The signals are closely related to the amount of fluorescein dye molecules present on the spot under examination. In evaluating the performance of this assay, each sample was tested ten times and based on the ratio of detected value to the cut-off (S/CO) values, the coefficient of variation was computed both between and within different lots.

When *P. falciparum* control panel was screened against commercially available RDTs, including Wondfo One-Step Malaria, the results showed that all samples with 5000 parasite/μl concentrations are within the detectable range of any good quality RDTs. The developed test showed a minimum limit of detection was 25 parasites/μl, which is far more sensitivity than the conventional immunochromatographic rapid diagnosis limit of 100 parasite/μl recommended by the WHO [20]. The results of performance evaluation for precision, stability and cross-interference tests showed that the indicators are in line with requirements. Interfering substances are a major cause of errors in the clinical application of *in vitro* diagnostic reagents. In this study, the negative reference samples with high concentrations of bilirubin, triglycerides, cholesterol, and rheumatoid factor were tested and the positive and negative coincidence rates were 100 %. Precision is an important measure of the performance of *in vitro* diagnostic reagents within and between batch assays. Precision assessment is an important basis for evaluating the effectiveness of the product. In clinical trials assessing parallel multi-centre study, the ratio of this newly developed reagent, as compared with reference reagents, was 100 % and the negative coincidence rate was 99.6 %. Three samples were detected positive by both the newly developed fluorescent HRP2 diagnostic test were however detected as negative samples when the reference diagnostic test kit was used. Further cross-examination by microscopy revealed that there are true positive specimens with parasite density of no more than 100 parasites/μl. This confirms the high level of accuracy of this novel development kit and an excellent correlation with the gold standard. It was also observed that the test had a high precision specificity for successfully detection of positive specimens and the possibility of producing less false negative or false positive results as compared to other immunochromatographic tests. Each test is estimated to cost at least one US dollar which make it affordable for developing and low-income countries. However, the test cannot adequately quantify the amount of HRP2 antigen in whole blood or serum samples. Further modifications are needed for future development of an HRP2 quantitative-based assay. Nonetheless, the very low detection limit of 25 parasites/μl make this technique a potential alternative to low sensitive RDTs in clinical settings.

## Conclusions

A sensitive, rapid and accurate immunofluorescence chromatography assay has been developed for the detection HRP2. The assay is an improved automated immunoassay, less time consuming and with higher sensitivity. The quick development of the assay is of great public health significance for prevention and control of the epidemic of falciparum infection and will provide important tools in prevention and control.
